# Adaptive Evolution of Geobacter sulfurreducens in Coculture with Pseudomonas aeruginosa

**DOI:** 10.1128/mBio.02875-19

**Published:** 2020-04-07

**Authors:** Lucie Semenec, Ismael A. Vergara, Andrew E. Laloo, Steve Petrovski, Philip L. Bond, Ashley E. Franks

**Affiliations:** a Department of Physiology, Anatomy and Microbiology, La Trobe University, Melbourne, Victoria, Australia; b Bioinformatics and Cancer Genomics, Research Division, Peter MacCallum Cancer Centre, Melbourne, Victoria, Australia; c Advanced Water Management Centre, The University of Queensland, Brisbane, Queensland, Australia; d Research Centre for Future Landscapes, La Trobe University, Melbourne, Victoria, Australia; Korea Advanced Institute of Science and Technology

**Keywords:** competition, evolution, mutualism, syntrophs

## Abstract

Interactions between microorganisms in mixed communities are highly complex, being either syntrophic, neutral, predatory, or competitive. Evolutionary changes can occur in the interaction dynamics between community members as they adapt to coexistence. Here, we report that the syntrophic interaction between Geobacter sulfurreducens and Pseudomonas aeruginosa coculture change in their dynamics over evolutionary time. Specifically, *Geobacter* sp. dominance increases with adaptation within the cocultures, as determined through quantitative PCR and fluorescence *in situ* hybridization. This suggests a transition from syntrophy to competition and demonstrates the rapid adaptive capacity of *Geobacter* spp. to dominate in cocultures with P. aeruginosa. Early in coculture establishment, two single-nucleotide variants in the G. sulfurreducens *fabI* and *tetR* genes emerged that were strongly selected for throughout coculture evolution with P. aeruginosa phenazine wild-type and phenazine-deficient mutants. Sequential window acquisition of all theoretical spectra-mass spectrometry (SWATH-MS) proteomics revealed that the *tetR* variant cooccurred with the upregulation of an adenylate cyclase transporter, CyaE, and a resistance-nodulation-division (RND) efflux pump notably known for antibiotic efflux. To determine whether antibiotic production was driving the increased expression of the multidrug efflux pump, we tested *Pseudomonas*-derived phenazine-1-carboxylic acid (PHZ-1-CA) for its potential to inhibit *Geobacter* growth and drive selection of the *tetR* and *fabI* genetic variants. Despite its inhibitory properties, PHZ-1-CA did not drive variant selection, indicating that other antibiotics may drive overexpression of the efflux pump and CyaE or that a novel role exists for these proteins in the context of this interaction.

## Abstract (precis)

*Geobacter* and *Pseudomonas* spp. cohabit many of the same environments, where *Geobacter* spp. often dominate. Both bacteria are capable of extracellular electron transfer (EET) and play important roles in biogeochemical cycling. Although they recently in 2017 were demonstrated to undergo direct interspecies electron transfer (DIET) with one another, the genetic evolution of this syntrophic interaction has not been examined. Here, we use whole-genome sequencing of the cocultures before and after adaptive evolution to determine whether genetic selection is occurring. We also probe their interaction on a temporal level and determine whether their interaction dynamics change over the course of adaptive evolution. This study brings to light the multifaceted nature of interactions between just two microorganisms within a controlled environment and will aid in improving metabolic models of microbial communities comprising these two bacteria.

## INTRODUCTION

*Geobacteraceae*, a family within the Deltaproteobacteria, predominantly reside in anaerobic environments, such as aquatic sediments, anaerobic wastewater, contaminated aquifers, and rice paddy soils, where they play a key role in the biogeochemical cycling of metals, sulfur, and carbon (1–8). Their relatively high abundance in these environmental niches (9–12) demonstrates their competitiveness in complex microbial communities. This stems from their metabolic flexibility which allows them to thrive under electron donor and nutrient-poor conditions (11, 13–16) and their adaptive ability to navigate toward metals via chemotaxis using flagella and pili (17). Furthermore, their enhanced ability to reduce insoluble electron acceptors of high redox potential via extracellular electron transfer (2, 6, 11), made possible by their hundreds of encoded *c*-type cytochromes and electrically conductive pili (18, 19), equip them with a competitive advantage to survive within mixed communities and conditions unfavorable for most organisms.

Although these physiological traits allow them to establish a dominant foothold in many of their environments, bacteria of this family, *Geobacteraceae*, are also adept at forming syntrophic interactions with other microorganisms. These can be formed via direct interspecies electron transfer (DIET), which utilizes *c*-type cytochromes and pili, hydrogen interspecies transfer-utilizing hydrogenases, or formate interspecies transfer utilizing formate dehydrogenase (20–23). Some of the studied interactions between *Geobacter* spp. and other species include G. sulfurreducens and G. metallireducens (23), G. sulfurreducens and Rhodoferax ferrireducens (24), *G. metallireducens* and Clostridium beijerinckii (25), G. sulfurreducens and Escherichia coli (26), G. sulfurreducens and Clostridium pasteurianum (27), and G. sulfurreducens and Wolinella succinogenes (28). Such studies aid in improving metabolic models of microbial communities.

Similar to *Geobacter* spp., Pseudomonas aeruginosa strains have also been found to participate in interspecies electron transfer, where P. aeruginosa endogenously produced phenazine shuttles electrons to electron-accepting bacteria (29, 30). Besides electron shuttling, *Pseudomonas* sp. phenazines are also known for their antibiotic properties, as are a multitude of other molecules they secrete (31, 32). P. aeruginosa and G. sulfurreducens have been found to coexist in many of the same environments, such as salt marsh estuaries, aquatic sediments, wastewater, and contaminated groundwater (33–38). Their potential for interaction, however, has only recently been investigated. Our recent adaptive evolution study on cocultures of G. sulfurreducens and P. aeruginosa found that several electron transfer processes were possible with the evolution of serially transferred cocultures, which progressively increased in fitness (39). Initially, DIET was the predominant form of syntrophic interaction. As they underwent 13 serial transfers, G. sulfurreducens upregulated its formate dehydrogenase (FdnG) and hydrogenase (HybA) enzymes, allowing for formate or hydrogen to be utilized as electron donors and suggesting an increase in its metabolic flexibility.

This study sought to understand the impact on the dynamics of interaction and the genetic basis of these observed changes. Fluorescence *in situ* hybridization (FISH) and quantitative PCR (qPCR) revealed an increase in the dominance of *Geobacter* spp. over coculture evolution, indicating a shift from syntrophy to competition. Whole-genome sequencing showed rapid selection for genetic variants in the *Geobacter fabI* and *tetR* genes. A frameshift mutation in *tetR* was coincident with significant upregulation of an adenylate cyclase-hemolysin transporter gene (*cyaE*) and a resistance-nodulation-division (RND) efflux pump (GSU0949), both located in a predicted TetR-regulated operon (40). TetR family transcriptional repressors are known for their role in regulating efflux pumps and transporters of antibiotics or toxic chemicals (41). Here, we find that G. sulfurreducens growth is inhibited by P. aeruginosa-derived phenazine-1-carboxylic acid (PHZ-1-CA). Selection of *tetR* and *fabI* variants is not explained by PHZ-1-CA. In agreement with this, we observe these variants to be present in *phz*-deficient cocultures. This suggests that other phenazine derivatives or nonphenazine antimicrobials secreted by P. aeruginosa drive the selection of these variants or, alternatively, there is an as-yet-unidentified role for RND efflux pump and adenylate cyclase upregulation in these cocultures.

## RESULTS

### *Geobacter* spp. increasingly dominate coculture populations through adaptation.

**FIG 1:**
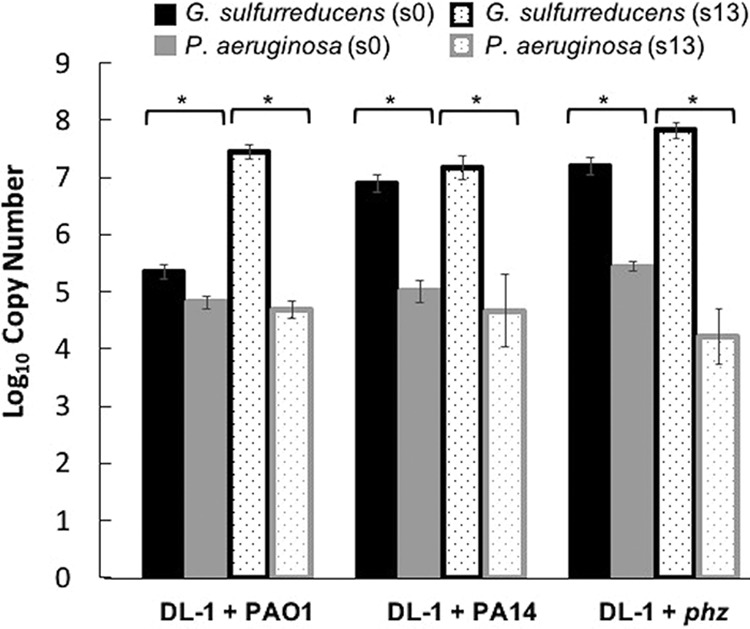
Proportion of G. sulfurreducens to P. aeruginosa in cocultures. Black and gray bars represent log copy numbers of DNA from early-stationary-phase cocultures using G. sulfurreducens-specific primers (Gsulf_F and Gsulf_R) and *Pseudomonas*-specific primers (Pse435F and Pse686R), respectively, via qPCR. Solid bars represent initial (s0) cocultures, and dotted bars represent adapted (s13) cocultures. *, *P* ≤ 0.05.

Previously, we observed that *Geobacter* spp. became more metabolically flexible with coculture adaptation. We also found that the abundance of *Geobacter* proteins was greater than that of *Pseudomonas* proteins in extracted coculture proteomes (39). To determine whether the increase in *Geobacter* metabolic flexibility changed coculture population dynamics over time, we directly quantified the abundance of each microbe in the population by performing qPCR on all cocultures (PAO1 + DL-1, PA14 + DL-1, and *phz* mutant + DL-1) for both initial (subculture 0 [s0]) and adapted (subculture 13 [s13]) cocultures. In both the initial and adapted cocultures, the *Geobacter* copy number significantly outnumbered that of *Pseudomonas* spp. (Fig. 1).

The dominance of G. sulfurreducens became more pronounced with adaptive evolution over 100 generations by s13, with the highest proportion of *Geobacter* to *Pseudomonas* spp. found in the adapted *phz* mutant + DL-1 cocultures (Table 1). This result shows that *Geobacter* spp. may be more likely to dominate *Pseudomonas* spp. in the absence of phenazine secretion and suggests a possible inhibitory role of phenazines on *Geobacter* spp. (see below). Overall bacterial abundance increased through successive coculture generations (Fig. S1), and *Geobacter* spp. became the most abundant community members by S13. This corresponds with its enhanced metabolic flexibility as it adapts (39), and hence its diminished reliance on *Pseudomonas* spp. for providing electron donors through DIET.

**TABLE 1:** Copy numbers of G. sulfurreducens and P. aeruginosa in cocultures as determined by qPCR

Coculture	Log copy no.		
	P. aeruginosa	G. sulfurreducens	G. sulfurreducens/P. aeruginosa ratio
PAO1 + DL-1 s0	6.6E+04	2.2E+05	3:1
PAO1 + DL-1 s13	4.8E+04	2.8E+07	589:1
PA14 + DL-1 s0	1.0E+05	7.7E+06	75:1
PA14 + DL-1 s13	4.7E+04	1.5E+07	316:1
*phz* mutant + DL-1 s0	2.8E+05	1.6E+07	57:1
*phz* mutant + DL-1 s13	1.7E+04	6.7E+07	3,980:1

10.1128/mBio.02875-19.1 FIG S1 Mean absorbance (OD_600_) reached at each serial transfer from s0 (initial coculture) to the 13th transfer s13 of G. sulfurreducens (DL-1) and P. aeruginosa (PAO1, PA14, and *phz* mutant) cocultures. Error bars represent standard error between three biological replicates. Download FIG S1, TIF file, 1.6 MB. Copyright © 2020 Semenec et al. 2020 Semenec et al. This content is distributed under the terms of the Creative Commons Attribution 4.0 International license.

**FIG 2:**
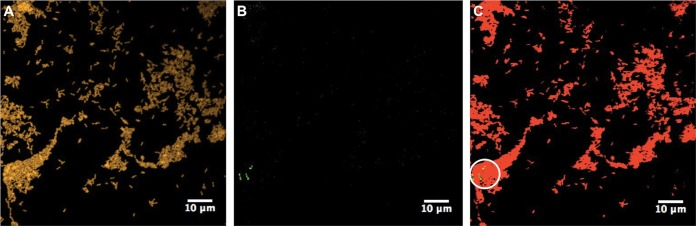
Proportion of G. sulfurreducens (DL-1) to P. aeruginosa (PAO1) in adapted (s13) cocultures via FISH. (A to C) All bacteria were probed with EUB338-ATTO633 probe (yellow) (A), the P. aeruginosa PAO1 strain was probed with PseaerA-ATTO488 probe (green) (B), and the G. sulfurreducens DL-1 strain was probed with GEO2-ATTO565 probe (red) (C). The white circle shows the localization of P. aeruginosa in flocs of G. sulfurreducens. Images are representative of triplicate samples taken during the early-stationary-growth phase of cocultures.

Due to the inability of qPCR to distinguish viable from nonviable cells (42), FISH was performed using probes (Table S1) targeting 16S rRNA, visualizing only the metabolically active population (43). FISH confirmed the dominant presence of *Geobacter* spp. (Fig. 2) in both the initial and adapted cocultures (Fig. S2 to S5). However, the *Pseudomonas* population as visualized by FISH was lower than that found by qPCR, and therefore, the majority of the *Pseudomonas* population identified by qPCR is likely to represent a subpopulation of either slow-growing/dormant cells, metabolically inactive cells, or extracellular DNA (44). Intriguingly, despite the larger population of *Geobacter* spp. and their increased metabolic flexibility over time, isolation of G. sulfurreducens from the cocultures where it was plated on Nutrient broth supplemented with acetate and fumarate (NBAF) agar under anaerobic conditions (see the Supplemental Methods) was not successful. Conversely, P. aeruginosa was easily isolated from the cocultures on LB agar under aerobic conditions (Fig. S6 and S7).

10.1128/mBio.02875-19.9 TEXT S1 Supplementary Methods. Download TEXT S1, DOCX file, 0.1 MB Copyright © 2020 Semenec et al. 2020 Semenec et al. This content is distributed under the terms of the Creative Commons Attribution 4.0 International license.

10.1128/mBio.02875-19.2 FIG S2 Proportion of G. sulfurreducens (DL-1) to P. aeruginosa (PA14) in adapted (s13) cocultures via FISH. (A to C) All bacteria were probed with EUB338-ATTO633 probe (yellow) (A), the P. aeruginosa PA14 strain was probed with PseaerA-ATTO488 probe (green) (B), and the G. sulfurreducens DL-1 strain was probed with GEO2-ATTO565 probe (red) (C). The white circle shows the localization of P. aeruginosa in flocs of G. sulfurreducens. Images are representative of triplicate samples taken during the early-stationary-growth phase of cocultures. Download FIG S2, TIF file, 1.3 MB. Copyright © 2020 Semenec et al. 2020 Semenec et al. This content is distributed under the terms of the Creative Commons Attribution 4.0 International license.

10.1128/mBio.02875-19.3 FIG S3 Proportion of G. sulfurreducens (DL-1) to P. aeruginosa (*phz* mutant) in adapted (s13) cocultures via FISH. (A to C) All bacteria were probed with EUB338-ATTO633 probe (yellow) (A), the P. aeruginosa *phz* mutant strain was probed with PseaerA-ATTO488 probe (green) (B), and the G. sulfurreducens DL-1 strain was probed with GEO2-ATTO565 probe (red) (C). The white circle shows the localization of P. aeruginosa in flocs of G. sulfurreducens. Images are representative of triplicate samples taken during the early-stationary-growth phase of cocultures. Download FIG S3, TIF file, 2.5 MB. Copyright © 2020 Semenec et al. 2020 Semenec et al. This content is distributed under the terms of the Creative Commons Attribution 4.0 International license.

10.1128/mBio.02875-19.4 FIG S4 Proportion of G. sulfurreducens (DL-1) to P. aeruginosa (PAO1) in adapted (s0) cocultures via FISH. (A to C) All bacteria were probed with EUB338-ATTO633 probe (yellow) (A), the P. aeruginosa *phz* mutant strain was probed with PseaerA-ATTO488 probe (green) (B), and the G. sulfurreducens DL-1 strain was probed with GEO2-ATTO565 probe (red) (C). The white circle shows the localization of P. aeruginosa in flocs of G. sulfurreducens. Images are representative of triplicate samples taken during the early-stationary-growth phase of cocultures. Download FIG S4, TIF file, 2.0 MB. Copyright © 2020 Semenec et al. 2020 Semenec et al. This content is distributed under the terms of the Creative Commons Attribution 4.0 International license.

10.1128/mBio.02875-19.5 FIG S5 Proportion of G. sulfurreducens (DL-1) to P. aeruginosa (*phz* mutant) in adapted (s0) cocultures via FISH. (A to C) All bacteria were probed with EUB338-ATTO633 probe (yellow) (A), the P. aeruginosa *phz* mutant strain was probed with PseaerA-ATTO488 probe (green) (B), and the G. sulfurreducens DL-1 strain was probed with GEO2-ATTO565 probe (red) (C). The white circle shows localization of P. aeruginosa in flocs of G. sulfurreducens. Images are representative of triplicate samples taken during the early-stationary-growth phase of cocultures. Download FIG S5, TIF file, 2.2 MB. Copyright © 2020 Semenec et al. 2020 Semenec et al. This content is distributed under the terms of the Creative Commons Attribution 4.0 International license.

10.1128/mBio.02875-19.6 FIG S6 Isolation of P. aeruginosa from cocultures with G. sulfurreducens on LB agar plates incubated at 30°C under aerobic conditions. (A) PA14 isolated from PAO1 + DL-1 cocultures. (B) PAO1 isolated from PA14 + DL-1 cocultures. (C) *phz* mutant from *phz* mutant + DL-1 cocultures. Download FIG S6, TIF file, 2.2 MB. Copyright © 2020 Semenec et al. 2020 Semenec et al. This content is distributed under the terms of the Creative Commons Attribution 4.0 International license.

10.1128/mBio.02875-19.8 TABLE S1 Primers and probes used for qPCR, FISH, and targeted sequencing in this study Download Table S1, DOCX file, 0.1 MB. Copyright © 2020 Semenec et al. 2020 Semenec et al. This content is distributed under the terms of the Creative Commons Attribution 4.0 International license.

### Genetic selection of *tetR* and *fabI* variants in early and late stages of coculture evolution.

**FIG 3:**
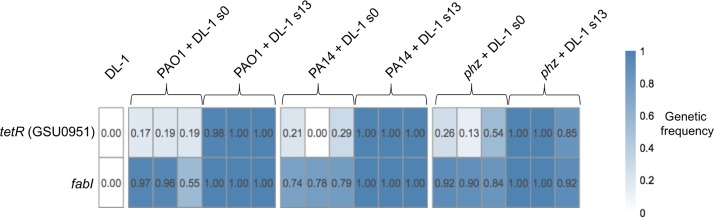
Genetic variants found in G. sulfurreducens from cocultures with P. aeruginosa initial (s0) and adapted (s13) via whole-genome sequencing. Heat map indicates the proportion of reads supporting the mutation in the specified gene, and numbers within each box represent the genetic frequency value. Three biological replicates per coculture were sequenced. The left column represents DL-1 pure culture in NBAF, and reads were aligned against the assembly of the Geobacter sulfurreducens PCA genome (NCBI assembly no. GCF_000007985.2).

Whole-genome sequencing of all cocultures and the original inoculum pure cultures was conducted to detect whether any mutations were selected for in the initial (s0) and adapted (s13) cocultures. Given the significantly lower abundance of P. aeruginosa in all cocultures, the genomic reads corresponding to P. aeruginosa were insufficient for statistically significant data analysis and variant calling. A comparison of the cocultures to the original inoculate of G. sulfurreducens revealed the presence of two genetic variants gained through coculture adaptation, a frameshift insertion in the *tetR* gene (GSU0951) and a missense single-nucleotide variant in the *fabI* gene (GSU1008), that were absent from the inoculate DL-1 genome (Fig. 3).

The *fabI* missense single-nucleotide variant was rapidly selected for early in coculture adaptation, possibly during lag phase, where an average of 83% of the reads across replicates from initially established cocultures had already acquired it (Fig. 3). Further selection occurred with evolution, whereby an average of 99% of the reads across replicates contained this mutation by the 13th transfer. This variant is characterized by a single nucleic acid substitution at genetic coordinate 1089002, resulting in a missense mutation that substitutes a serine for a proline at amino acid position 122. This gene encodes FabI, an enoyl-acyl-carrier protein (enoyl-ACP) reductase which catalyzes the last step in fatty acid biosynthesis (45, 46). FabI is a target for several antibiotics, including diazaborine (47), isoniazid (48), and triclosan (49), and a single amino acid substitution within it is sufficient to confer resistance to these antibiotics (48, 50–54).

Early selection was also observed for a genomic variant of the GSU0951 (here referred to as *tetR*) gene, which was found in an average of 22% of the initial coculture reads across replicates. Similar to the *fabI* variant, the *tetR* variant was strongly selected for through coculture adaptation, becoming present in an average of 98% of the adapted coculture reads. This variant was characterized by a single-nucleotide polymorphism (SNP) involving an insertion at genetic locus 1023986. This SNP resulted in a frameshift mutation, changing the identities of 43 amino acids downstream of this position, followed by an early stop codon, leading to premature truncation of the protein. The absence of this protein from all coculture proteomes suggests that this mutation is likely deleterious. Although G. sulfurreducens pure cultures grown in syntrophic NB(formate + fumarate) medium were not evolved through serial transfers due to their poor growth in this medium, we recently observed that adaptively evolved PilA-deficient mutants of G. sulfurreducens (via 13 serial transfers) in NB(formate + fumarate) medium do not acquire any mutations in *tetR* or *fabI* (55). Therefore, these two mutations appear to be specifically acquired in response to the presence of P. aeruginosa.

### Evidence for TetR regulation of CyaE and an RND efflux pump.

**FIG 4:**
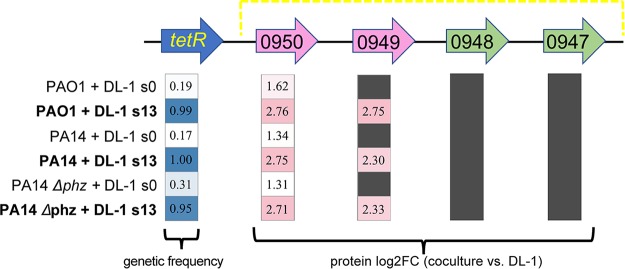
Correlation between G. sulfurreducens *tetR* SNP mutation frequency and downstream operon protein abundance. Blue cells indicate the average frequency of genetic reads across replicates obtained from whole-genome sequencing (WGS) that contain the single-nucleotide insertion in GSU0951 (*tetR*). Pink cells indicate the log_2_ fold change (log_2_FC) of protein abundance in the cocultures versus DL-1 pure cultures. Gray cells indicate proteins absent from the proteome. The yellow dashed box indicates a previously predicted operon (40). Genes depicted by pink arrows represent those likely under the control of TetR (in blue), as confirmed by this study. Genes depicted by green arrows represent those absent from the proteomes.

*tetR* encodes a predicted transcriptional regulator of the TetR family, the members of which are known to behave as repressors of transcriptional activity (40). TetR is situated upstream of a predicted operon composed of genes *GSU0950* and *GSU0949*, which putatively encode an outer membrane adenylate cyclase transporter (CyaE) and an RND efflux pump, respectively, and predicted ABC transporter genes, *GSU0948* and *GSU0947* (40). As TetR is predicted to negatively regulate this operon, we looked at the protein abundances of these gene products within our proteomics data previously obtained by sequential window acquisition of all theoretical spectra-mass spectrometry (SWATH-MS) (39). Both GSU0950 (herein referred to as CyaE) and GSU0949 (RND family efflux pump) were found in significantly higher abundances in cocultures than in G. sulfurreducens pure cultures. As the genetic frequency of the *tetR* variants increased from 22% (range, 17 to 31%) to 98% (range, 95 to 100%) with adaptive evolution, the log_2_ fold change (log_2_FC) (coculture versus DL-1) of CyaE protein abundance increased from an average of 1.4 (range, 1.31 to 1.62) to 2.7 (range, 2.71 to 2.76) (Fig. 4). Similarly, GSU0949 increased in abundance, with adaptation from initially absent to a log_2_FC of 2.46 (range, 2.30 to 2.75). Interestingly, GSU0948 and GSU0947 were not found in the extracted proteomes, suggesting a lack of control of these genes by TetR under the conditions of this study.

### Antimicrobial activity of PHZ-1-CA does not select for *tetR* and *fabI* genetic variants.

Due to the ability of RND efflux pumps to export antibiotics like *Pseudomonas* phenazines (56) and our finding that *Geobacter* dominance over *Pseudomonas* spp. was highest in the absence of phenazines, we sought to test the antimicrobial activity of phenazines on G. sulfurreducens. We measured the MIC of PHZ-1-CA, a precursor to PYO, phenazine-1-carboxamide, and 1-hydroxyphenazine (57), for G. sulfurreducens in adapted cocultures versus that of the G. sulfurreducens DL-1 wild type (not previously exposed to P. aeruginosa). The MIC of cocultures with PAO1 and PA14 was 250 mg/liter (Table 2), whereas the MIC of *phz* mutant cocultures was 125 mg/liter after 38 days. The MIC of the DL-1 wild type was 125 mg/liter, the same as for the *phz* mutant coculture, and after one serial transfer (s1), it increased to 250 mg/liter. This result confirms a partial inhibitory activity of PHZ-1-CA on G. sulfurreducens and suggests that the various phenazine derivatives may be secreted by the PAO1 + DL-1 and PA14 + DL-1 cocultures given their higher MICs over that of the *phz* mutant + DL-1 coculture. Since the *tetR* and *fabI* mutations were acquired already in s0 cocultures, we assessed whether DL-1 s1 acquired mutations in *tetR* or *fabI* after one serial transfer in PHZ-1-CA. Targeted next-generation sequencing of these genes at high coverage (∼27,000×) found no variants with an allelic frequency of 2% or greater that were absent in G. sulfurreducens not treated with PHZ-1-CA. This result indicates that *tetR* and *fabI* mutations are not selected for due to the presence of PHZ-1-CA in particular. This suggests that other P. aeruginosa antibiotics and phenazine derivatives, such as anthranilate, PYO, and 1-hydroxyphenazine, may be driving the selection of these mutations. In agreement with this, PHZ-1-CA was below the detection limits in all cocultures (see Supplemental Methods), as previously observed for P. aeruginosa PAO1 pure cultures grown in minimal medium (57).

**TABLE 2:** Phenazine-1-carboxylic acid MIC after 38 days

Culture	MIC (mg/liter)
PAO1 + DL-1 s13	250
PA14 + DL-1 s13	250
*phz* mutant + DL-1 s13	125
DL-1	125
DL-1 s1	250^*a*^
PAO1	500
PA14	500
*phz* mutant	500

a MIC after 30 days.

## DISCUSSION

*Geobacter* and *Pseudomonas* spp. are electrochemically active bacteria found to cohabit many of the same environments of ecological significance, including contaminated sites and wastewater streams (35, 38). In this study, assessment of their adaptive evolution using serial transfers *in vitro* enabled observations of real-time interaction dynamics and survival strategies, something not easily acquired through *in situ* experiments given the complexity of their environments and the presence of other community members.

For all three *Pseudomonas* strains, PAO1, PA14, and the *phz* mutant, tested in cocultures with G. sulfurreducens, the *Geobacter* sp. population was dominant. Throughout time, *Geobacter* sp. dominance increased with coculture evolution. The increasing dominance of *Geobacter* spp. resembles a transition from initial syntrophy to a competitive interaction, where *Geobacter* spp. may be benefiting at the cost of *Pseudomonas* spp. Interestingly, isolation of G. sulfurreducens from the cocultures was not possible despite its dominance, suggesting a reliance on *Pseudomonas* spp. This was the case for both initial s0 and adapted s13 cocultures. Not only was G. sulfurreducens more dominant than was P. aeruginosa, but it also appeared to be more metabolically active, as indicated by FISH. Bacteria enter dormancy as a bet-hedging strategy and in response to various environmental factors, including nutrient limitation (58), competition (59), and antibiotics (60). Alternatively, their growth can be inhibited through contact-dependent inhibition or predation as an extreme response to limited nutrients (61, 62). It is yet unclear whether G. sulfurreducens inhibits P. aeruginosa growth, which would suggest possible predation, or whether P. aeruginosa ushers itself into dormancy in order to protect itself from the limited and competitive conditions (63, 64). This warrants further investigation, especially given the close relation of *Geobacter* spp. to the prokaryotic predators Bdellovibrio spp. that reside in similar environments and can prey on various *Pseudomonas* species (65, 66). Given that *Geobacter* and *Pseudomonas* spp. coexist in many oligotrophic and contaminated environments where *Pseudomonas* spp. are often less abundant than *Geobacter* spp. (33, 35, 37), we believe that their coculture interaction dynamics observed in this study may have ecological relevance.

This study provides the first account for selection of *tetR* and *fabI* mutations in *Geobacter* spp. as a response to coculture evolution with P. aeruginosa. The consistent selection for *tetR* and *fabI* variants observed across all three cocultures and three biological replicates strongly suggests an adaptive evolutionary role of these mutations to the fitness of *Geobacter* spp. in cocultures with P. aeruginosa. Furthermore, the observation that increased genetic frequency of the *tetR* frameshift variant mirrored the increased expression of downstream genes, GSU0950 and GSU0949, is in agreement with the predicted regulatory role of *tetR* within the TetR operon (40). The results described here show the propensity of G. sulfurreducens to undergo rapid adaptive evolutionary changes that may play a role in protection against competition with stresses created by P. aeruginosa, as *Geobacter* spp. become more metabolically versatile during coculture adaptation (39). Strong and rapid selection driven by a limited number of mutations has been observed in other work, where a single-nucleotide mutation of a regulatory gene confers rapid adaptive resilience to stress (23, 67, 68).

TetR-regulated RND efflux transporters are important for antibiotic or toxic chemical efflux (41). *Pseudomonas* spp. are known to produce a vast array of antibiotics, including phenazines and quinolones, that have various antimicrobial properties on different bacterial species (31, 32). Recently, it was shown that P. aeruginosa itself uses the RND family efflux protein complex MexGHI-OpmD to secrete the PYO intermediate 5-methylphenazine-1-carboxylate (5-Me-PCA) and thus enables self-resistance to this highly reactive compound (56). Besides phenazines, this same efflux pump can also export the toxic *Pseudomonas* quinolone signal (PQS) precursor anthranilate (69), the antibiotic norfloxacin, and the toxic dye acriflavine (70), which demonstrates the wide range of substrates for this efflux pump. We found that one serial transfer of unadapted *Geobacter* spp. in PHZ-1-CA resulted in an increased MIC but did not correspond with the selection of *tetR* and *fabI* variants. Hence, it is likely that posttranscriptional or posttranslational modifications allowed for adaptation to *Pseudomonas* sp. PHZ-1-CA. In agreement with this, coculture of *Geobacter* spp. with a phenazine-deficient *Pseudomonas* mutant still resulted in the selection of *tetR* and *fabI* mutations. In future work, a comprehensive panel of P. aeruginosa antibiotics needs to be screened for their potential to drive selection of the *tetR* and *fabI* variants in order to rule out whether these variants arise as an antimicrobial response. In addition to GSU0949 RND efflux pump upregulation, the adenylate cyclase transporter CyaE (GSU0950) is also upregulated in response to adaptation with P. aeruginosa. Adenylate cyclases catalyze the synthesis of the second messenger, cAMP, and are commonly attributed to their toxic effects on eukaryotic hosts, as in the case of Bordetella pertussis and Vibrio cholerae (71). Their role in interactions with P. aeruginosa, however, requires further investigation, especially given the inability to isolate G. sulfurreducens from P. aeruginosa in the cocultures.

This work demonstrates the complex interplay between syntrophy and competition that can occur between just two microbial species in a defined medium. Microbial communities can adapt mutually beneficial interactions often involving cross-feeding or exchange of electrons (72). Such interactions have commonly been found in methanogenic communities where syntrophy between methanogens, fermentative bacteria, and acetogens takes place (73). Furthermore, DIET allows the exchange of reducing equivalents between syntrophic microbial partners and has been found to occur in various environments and under lab culture conditions (2, 12, 23, 74). Although DIET appears as an initial form of syntrophy between G. sulfurreducens and P. aeruginosa, this interaction transitions to competition as G. sulfurreducens increases its utilization of formate. Our finding corresponds well with previous hypotheses that in a majority of cases, adaptation of two culturable microbial species leads to competitive rather than cooperative interactions, even in a medium designed for syntrophy (75). In this coculture, competition eventually supersedes syntrophy as *Geobacter* spp. adapt to utilize hydrogen and formate as electron donors. This can be a valuable lesson when evaluating the immense array of interactions that occur at the microbial community level in natural environments.

## MATERIALS AND METHODS

### Strains and media.

G. sulfurreducens DL-1 (PCA strain, ATCC 51573) was grown under strictly anaerobic conditions in NB supplemented with acetate and fumarate (NBAF) medium, as previously described (76), except no resazurin was added. For growth of the pure culture, 20 mM acetate and 40 mM fumarate were used as the electron donor and acceptor, respectively. P. aeruginosa PAO1, PA14, and PA14 *Δphz1 Δphz2* (here referred to as the *phz* mutant) (57) strains were grown under strictly anaerobic conditions in NB medium containing 20 mM formate and 20 mM nitrate as the electron donor and acceptor. The two strains of P. aeruginosa differ in their phenazine production under different conditions, where PAO1 produces 4-fold higher pyocyanin (PYO) than does PA14 in medium that mimics the cystic fibrotic lung (77), while PA14 produces 10-fold higher pyocyanin PAO1 in Luria-Bertani (LB) medium (57). For growth of the cocultures, 20 mM formate and 40 mM fumarate were provided as the electron donor and acceptor, the former of which is preferentially utilized by *Pseudomonas* spp., and the latter of which is only utilized by *Geobacter* spp. To initiate s0 cocultures, each bacterial species from pure cultures grown to late-log phase was inoculated at a concentration of 3.4 × 10^7^ cells/ml as determined via a hemocytometer to ensure equal representation of *Geobacter* and *Pseudomonas* spp. when establishing the coculture. Adaptation of the cocultures involved transferring 1% of culture to fresh NB(formate/fumarate) medium once they reached stationary phase, the growth stage when P. aeruginosa begins producing phenazines (57), as determined by the optical density at 600 nm (OD_600_). The cultures were adapted to up to 13 transfers. The initial culture is referred to here as subculture 0 (s0), and the 13th transfer culture is called subculture 13 (s13). In total, 13 transfers were made, and stocks of cultures were flash frozen in liquid nitrogen and stored at −80°C.

### SWATH-MS proteomics.

All cell harvesting, protein extraction, proteomics, and statistical analyses were performed as previously reported (39).

### Genomic DNA extractions and next-generation sequencing.

Each coculture comprised three biological replicates. For each biological replicate, there were an additional eight pooled technical replicate cultures. One milliliter of culture was harvested from each replicate at early stationary phase and centrifuged at 14,000 relative centrifugal force (rcf) for 10 min. The supernatant was removed, and cell pellets were snap frozen in liquid nitrogen and stored at −80°C until genomic DNA extraction. Genomic DNA extractions were done using the Isolate II genomic DNA kit from Bioline, according to the manufacturer’s instructions. DNA concentrations were measured on the Implen NanoPhotometer P330, and samples were normalized to the same concentration before pooling all technical replicates to obtain an even representation of each replicate. Whole-genomic libraries were made with the Nextera XT kit (Illumina Inc., Australia), and library concentration and mean fragment lengths were measured using a Qubit fluorometer (Invitrogen) and Bioanalyzer (Agilent Technologies). Next-generation sequencing with 75-bp paired-end reads and 150 cycles in MID-output mode was performed on the Illumina NextSeq 500 platform by the Australian Genome Research Facility (Parkville, Victoria, Australia).

### Assigning reads in sequenced coculture genomes.

Adapter sequences were trimmed from paired-end reads using Cutadapt v1.13 (78), with a required minimum length (m) of 30 and a quality cutoff (q) of 15. Trimmed reads were aligned against the GCF_000007985.2 assembly of the Geobacter sulfurreducens PCA genome, the GCF_000006765.1 assembly of Pseudomonas aeruginosa PAO1, and the GCF_000404265.1 assembly of Pseudomonas aeruginosa PA14 using SMALT v0.7.6 (http://www.sanger.ac.uk/science/tools/smalt-0). The SMALT index for each genome was built using a k-mer (k) size of 11 and a step size (s) of 1. For each coculture of Geobacter sulfurreducens PCA and a *Pseudomonas* strain, the alignment of paired-end reads against each reference genome was used to categorize each read pair using SAMtools (78–81) as properly mapped (-f 0 × 2), or not (-F 0 × 2) distinguishing reads unmapped (-f 0 × 4). Given the two genomes (here referred to as species A and B), reads were assigned as originating from genome A if they were (i) mapped to genome A and unmapped to genome B, or (ii) mapped to both genomes, with a sum of the SMALT alignment for both reads in the pair in genome B being less than 80% of the sum of the alignment score in genome A. Read pairs not fulfilling these criteria or with a mapping quality score of less than 30 were excluded from further analysis. Using the above-described categorization, reads assigned to each genome were marked for duplicates with picard v2.9.3 (http://broadinstitute.github.io/picard/) and realigned with the Genome Analysis Tool kit (GATK) v3.5-0 (80). Single-nucleotide variants (SNVs) and small insertions/deletions were called using the HaplotypeCaller function of GATK, with an indel size (indelSizeToEliminateInRefModel) of 20, a minimum mapping quality (mmq) score of 30, and ploidy of 2 to allow for detection of mutations occurring in a subset of the cell population. The impact of mutations on protein-coding transcripts was assessed using CooVar v0.07 (81).

### Harvesting for FISH and cell fixation.

During harvesting of cocultures at early stationary phase, as described above, 2 ml of each culture was harvested for FISH. Harvesting at early stationary phase for pure cultures of each bacteria was also performed and used for positive- and negative-control tests. Cells were pelleted at 4,000 rcf for 10 min, resuspended in 1× phosphate-buffered saline (PBS), and then fixed in 4% paraformaldehyde for 12 h at 4°C. The cells were pelleted and washed two times in 1× PBS, followed by resuspension in a 1:1 ratio of 100% ethanol with 1× PBS, after which they were stored at –20°C until hybridization was performed within 1 month of storage.

### Hybridization and microscopy.

Hybridization was carried out according to the protocol by Erhart et al. (82), with minor modifications. Hybridizations were carried out at 46°C for 2 h in a humidity chamber using a hybridization buffer, as previously described (82); after testing a range of formamide concentrations, a 40% formamide concentration was found to give the most specific binding of the probes used, allowing distinct detection of each bacterial species compared to negative controls with no probe bound. Following hybridization, a 25-min wash step at 48°C was performed in a washing buffer, as previously described (82). The EUB338 FISH probe (83), which labels most bacteria, was used as a positive label for both G. sulfurreducens and P. aeruginosa and 5′ labeled with ATTO633. P. aeruginosa FISH probe (84) targeting the 16S rRNA was 5′ labeled with ATTO488. The G. sulfurreducens (23) FISH probe targeting the 16S rRNA was 5′ labeled with ATTO565. Labeled probes were obtained from Integrated DNA Technologies Pte. Ltd. (IDT, Baulkham Hills, NSW, Australia). All cells were also stained with the DNA binding dye 4′,6-diamidino-2-phenylindole (DAPI) to visualize all cells and ensure there were not any contaminant microorganisms in the cocultures not belonging to P. aeruginosa or G. sulfurreducens. Cells were visualized on a Zeiss confocal LSM780 microscope with a 63× and 100× oil immersion objective (Carl Zeiss MicroImaging GmbH, Germany) at the LIMS BioImaging Facility (La Trobe University, Australia).

### Cloning standards and quantitative PCR.

Gene fragments of G. sulfurreducens and P. aeruginosa 16S rRNA were PCR amplified using *Taq* DNA polymerase (Qiagen, Australia) and the primers listed in Table S1. G. sulfurreducens has a 16S rRNA gene copy number of 2, and P. aeruginosa has a copy number of 4. The reaction conditions were as follows: 94°C for 2 min, followed by 32 cycles at 94°C for 1 min, 60°C for 1 min, and 72°C for 1.5 min. The fragments were then cloned into the TOPO TA Cloning kit for subcloning with One Shot TOP10 chemically competent E. coli cells (Invitrogen), according to the manufacturer’s instructions for the transformation of One Shot Mach-1 competent cells. The transformed clones were picked for plasmid miniprep using the PureYield plasmid miniprep system (Promega, Australia). Plasmids pLS1 and pLS2, containing cloned P. aeruginosa 16S rRNA and G. sulfurreducens 16S rRNA gene fragments, respectively, were confirmed with sequencing by the Australian Genome Research Facility (AGRF). Quantitative PCR was performed to determine the ratio of G. sulfurreducens to P. aeruginosa in the cocultures. This quantification was done using the cloned 16S rRNA gene fragments to produce a standard curve. Their concentration was determined on the Implen NanoPhotometer P330, from which the copy number was calculated, and a 5-fold dilution series was made (10^9^ to 10^2^ genes/μl) to generate a standard curve. The primers (Table S1) used for the quantification of G. sulfurreducens gene copy numbers were Gsulf_F and Gsulf_R, and Pse435F and Pse686R were used for P. aeruginosa quantification, generating amplicons of 108 bp and 251 bp, respectively. Reactions were performed in a total volume of 20 μl with 3.3 μl SensiFAST SYBR and fluorescein mix (Bioline, Australia), 133 nM each primer, and 2 μl of DNA template. The qPCR cycle settings started with an initial denaturation at 94°C for 3 min, followed by 40 cycles of 94°C for 10 s and 60°C (G. sulfurreducens) or 58°C (P. aeruginosa) for 30 s, with a melting curve of 60°C for 30 s and temperature increasing to 95°C, with data readings at increments of 0.5°C.

### Phenazine-1-carboxylic acid MIC.

The MICs of PHZ-1-CA were determined using a broth dilution method where 2-fold serial dilutions (from 500 mg/liter to 2 mg/liter) of PHZ-1-CA (Merck) were made in anaerobic Hungate tubes and inoculated with cocultures (DL-1 + PAO1, DL-1 + PA14, and DL-1 + *phz* mutant) and pure cultures of DL-1, PAO1, PA14, and the *phz* mutant. The dilutions were made in 10% ethanol of either NB(formate + fumarate) medium (for cocultures), NBAF medium (for DL1 pure cultures), or NB(formate + nitrate) medium (for PAO1, PA14, and *phz* mutant pure cultures). Positive-control and ethanol control tubes were also inoculated with culture and contained medium only or 10% ethanol medium, respectively, with no PHZ-1-CA. Once the cultures reached early stationary phase, they were diluted to a turbidity equivalent to 0.5 McFarland standard (OD_625_, 0.1) corresponding to ∼1 × 10^8^ to 2 × 10^8^ cells (85), and 1% of the culture was added to the dilution series tubes. The tubes were incubated at 30°C for 30 to 38 h. The MIC was established as the lowest concentration of PHZ-1-CA that inhibited growth.

### Targeted sequencing of *tetR* and *fabI*.

Targeted next-generation sequencing of the *tetR* and *fabI* regions was performed on G. sulfurreducens DL-1 pure cultures grown in phenazine-1-carboxylic acid (Merck) at various concentrations for MIC testing. Primers were designed to amplify ∼350-bp regions of each gene and contained Illumina overhang adapter sequences (Table S1). For each ∼1-kb gene, three primer pairs were used to cover the entire length of the gene. Genomic DNA extractions were done using the Isolate II genomic DNA kit from Bioline, according to the manufacturer’s instructions. DNA concentration was measured on the Implen NanoPhotometer P330, and samples were normalized to 5 ng/μl and used to generate amplicon libraries with the MiSeq reagent kit V3 (Illumina, Inc., Australia). The *tetR* and *fabI* primers with appropriate Illumina adapters for the forward and reverse primers (Table S1) were used to generate amplicons according to the 16S rRNA metagenomic sequencing library preparation protocol (part no. 15044223 rev. B; Illumina). PCR cleanup was performed using AMPure XP beads, following the manufacturer’s instructions, and subsequent PCR was performed to add Illumina flow cell adapters and Nextera XT indices. DNA concentration and mean fragment lengths were measured using a Qubit fluorometer (Invitrogen) and Agilent 4200 TapeStation (Agilent Technologies). Next-generation sequencing with 300-bp paired-end reads and 600 cycles was performed on the Illumina MiSeq platform.

### Data availability.

All raw sequences are deposited in the Sequence Read Archive under BioProject number PRJNA544640 and BioSample numbers SAMN11792103 to SAMN11792109. The mass spectrometry proteomics data have been deposited to the ProteomeXchange Consortium via the PRIDE partner repository (86) with the data set identifier PXD013990.

10.1128/mBio.02875-19.7 FIG S7 Single-colony PCR of P. aeruginosa isolated from cocultures on LB agar using Gsulf and Pse primers run on a.1.5% agarose gel. Lane 1, 100-bp NEB ladder; lanes 2 to 4, PAO1 isolates from cocultures (replicates 1 to 3) for first transfer on LB (t1); lanes 5 to 7, PA14 isolates from cocultures (t1); lanes 8 to 10, *phz* mutants from cocultures (t1); lanes 11 to 13, PAO1 isolates from cocultures for second transfer on LB (t2); lanes 14 to 16, PA14 isolates from cocultures (t2); lanes 17 to 19, *phz* mutant isolates from cocultures (t2); lane 20, DL-1 negative control (water plus Gsulf primers); lane 21, P. aeruginosa negative control (water plus Pse primers); lane 22, DL-1 positive control (DL-1 genomic DNA [gDNA] plus Gsulf primers); lane 23, P. aeruginosa positive control (PAO1 gDNA plus Pse primers). Download FIG S7, TIF file, 2.4 MB. Copyright © 2020 Semenec et al. 2020 Semenec et al. This content is distributed under the terms of the Creative Commons Attribution 4.0 International license.
