# Supplementary material for: Adaptive Evolution of Geobacter sulfurreducens in Coculture with Pseudomonas aeruginosa
Source: mBio. 2020 Apr 7;11(2):e02875-19. doi: 10.1128/mBio.02875-19 (PMC7157779; doi:10.1128/mBio.02875-19)
Supplement: TEXT S1 [file mBio.02875-19-s0001.docx]

**Supplementary Methods**

*Isolation from cocultures*

*P. aeruginosa* was isolated from *G. sulfurreducens* by plating cocultures onto Luria-Bertani (LB) agar plates and transferring single colonies three times. Plates were incubated aerobically at 30°C. Single colony PCR was performed using 16S rRNA primers specific to *G. sulfurreducens* (Gsulf – 108 bp amplicon) and the same colony was used for single colony PCR using *Pseudomonas* specific primers (Pse – 251 bp amplicon) (Table S1) to confirm isolation of *P. aeruginosa*. Equivalent volumes of each PCR product were loaded into the same lane on a 1.5% TAE agarose gel stained with SYBR Safe to inspect whether the colony contained each species (Figure S5). PCR of negative and positive controls (containing *G. sulfurreducens* genomic DNA and *P. aeruginosa* genomic DNA) for each primer pair was also performed. To isolate *G. sulfurreducens* from *P. aeruginosa*, cocultures were plated on NBAF agar plates poured anaerobically and incubated at 30°C in an anaerobic glove bag under an atmosphere of N_2_-CO_2_-H_2_ (85:10:5).

*Measurement of phenazine-1-carboxylic acid*

*In vitro* production of phenazine-1-carboxylic acid in adapted s13 cocultures was measured via HPLC (ACS Laboratories, Australia). Cocultures were grown to early stationary phase and culture filtrate was collected by filter sterilization with a 0.22 µm filter (Millipore) and stored at 4°C until HPLC was performed.
