# Supplementary material for: Adaptive Evolution of Geobacter sulfurreducens in Coculture with Pseudomonas aeruginosa
Source: mBio. 2020 Apr 7;11(2):e02875-19. doi: 10.1128/mBio.02875-19 (PMC7157779; doi:10.1128/mBio.02875-19)
Supplement: TABLE S1 [file mBio.02875-19-st001.docx]

**Table S1 –** Primers and probes used for qPCR, FISH and targeted sequencing in this study

| Name | Sequence | Method | Amplicon Size (bp) | Reference |
| --- | --- | --- | --- | --- |
| Gsulf_F | 5’-CCAGCTACGCCTACTTCTTCTTT-3’ | qPCR | 108 | (Summers et al 2010) |
| Gsulf_R | 5’-AAGCTGTGGTTCAGGAGGTATTT-3’ | qPCR |  | (Summers et al 2010) |
| Pse435F | 5’- ACTTTAAGTTGGGAGGAAGGG-3’ | qPCR | 251 | (Bergmark et al 2012) |
| Pse686R | 5’-ACACAGGAAATTCCACCACCC-3’ | qPCR |  | (Bergmark et al 2012) |
| GEO2 | 5’-GAAGACAGGAGGCCCGAAA-3’ | FISH | NA | (Summers et al 2010) |
| PseaerA | 5’- GGTAACCGTCCCCCTTGC-3’ | FISH | NA | (Hogardt et al 2000) |
| EUB338 | 5’-GCTGCCTCCCGTAGGAGT-3’ | FISH | NA | (Amann et al 1990) |
| Tet_F1 | 5'-TCGTCGGCAGCGTCAGATGTGTATAAGAGACAGTTCTCCATTGACACGCCCC-3' | targeted sequencing | 323 | (this study) |
| Tet_R1 | 5'-GTCTCGTGGGCTCGGAGATGTGTATAAGAGACAGGCCAGGTTGATTTCCAGCTT-3' | targeted sequencing |  | (this study) |
| Tet_F2 | 5'-TCGTCGGCAGCGTCAGATGTGTATAAGAGACAGCATCTATCGTCACTTCGGCG-3' | targeted sequencing | 320 | (this study) |
| Tet_R2 | 5'-GTCTCGTGGGCTCGGAGATGTGTATAAGAGACAGGCCATTTTCTCCGCCAGTC-3' | targeted sequencing |  | (this study) |
| Tet_F3 | 5'-TCGTCGGCAGCGTCAGATGTGTATAAGAGACAGCCGCTTCATTTTCTCCGAGG-3' | targeted sequencing | 390 | (this study) |
| Tet_R3 | 5'-GTCTCGTGGGCTCGGAGATGTGTATAAGAGACAGCATTTGCTCACTTTTGGGGTAA-3' | targeted sequencing |  | (this study) |
| Fab_F1 | 5'-TCGTCGGCAGCGTCAGATGTGTATAAGAGACAGCAGGTGACAGCATCCGGTA-3' | targeted sequencing | 340 | (this study) |
| Fab_R1 | 5'-GTCTCGTGGGCTCGGAGATGTGTATAAGAGACAGAAACTGCCGCAATGTCGT-3' | targeted sequencing |  | (this study) |
| Fab_F2 | 5'-TCGTCGGCAGCGTCAGATGTGTATAAGAGACAGATTCTCCCCTGCGACGTT-3' | targeted sequencing | 373 | (this study) |
| Fab_R2 | 5'-GTCTCGTGGGCTCGGAGATGTGTATAAGAGACAGTAGCTCGGGAAAACCTTCTG-3' | targeted sequencing |  | (this study) |
| Fab_F3 | 5'-TCGTCGGCAGCGTCAGATGTGTATAAGAGACAGGGGGAGCATTATCACCCTTT-3' | targeted sequencing | 492 | (this study) |
| Fab_R3 | 5'-GTCTCGTGGGCTCGGAGATGTGTATAAGAGACAGCGTGCAGCTGTTTGATTGAA-3' | targeted sequencing |  |  |

*Illumina adapter sequences are represented in blue text.
